# Isolation and characterization of *Listeria monocytogenes* among women attending Jimma University medical center, Southwest Ethiopia

**DOI:** 10.1186/s12879-021-06254-w

**Published:** 2021-06-12

**Authors:** Lencho Girma, Alene Geteneh, Demisew Amenu, Tesfaye Kassa

**Affiliations:** 1 Department of Medical Laboratory Science, Mizan Aman College of Health Sciences, Aman, Ethiopia; 2 Department of Medical Laboratory Science, College of Health Sciences, Woldia University, Woldia, Ethiopia; 3 Department of Obstetrics and Gynecology, School of Medicine, Jimma University, Jimma, Ethiopia; 4 School of Medical Laboratory Sciences, Jimma University, Jimma, Ethiopia

**Keywords:** Women, *Listeria monocytogenes*, Antimicrobial susceptibility, Biofilm, Ethiopia

## Abstract

**Background:**

*Listeria monocytogenes* (LM) has come to be a major public health issue of at-risk groups, causing high morbidity and mortality. Despite this data, studies are very limited in developing countries like Ethiopia. Thus, we aimed to isolate and characterize LM in terms of antibiogram and biofilm formation among pregnant women with fever, women with a history of spontaneous abortion, women with a history of fetal loss, and women with preterm delivery at Jimma University Medical Center (JUMC), southwest Ethiopia.

**Methods:**

A cross-sectional study was done among 144 women from June to August 2019. Isolates were tested for antibiotic susceptibility and biofilm formation using disc diffusion and microtiter plate method, respectively. Data were collected using a structured questionnaire, entered into Epidata 3.1 and logistic regression was done by SPSS v25.0.

**Results:**

LM was isolated in 8 (5.56%) of 144 screened women. The isolation rate of LM was relatively higher among women with a history of fetal loss (9.7%), followed by women with preterm delivery (6.25%). One of the six cord blood was positive for LM, indicating that the transplacental transmission rate at JUMC was 16.7%. More than 2% of women with an ongoing pregnancy were found to have LM septicemia, which could hurt their fetus. All of the isolates tested were susceptible to Ampicillin. However, all of the isolates were resistant to Penicillin and Meropenem and were biofilm producers.

**Conclusions:**

The high magnitude of pregnancy-related listeriosis in the current study setting appears that implementation of educational programs targeting risk reduction and more studies to identify sources of LM are warranted. The choice of antibiotics should be after susceptibility testing.

## Introduction

*Listeria monocytogenes (LM),* a causative agent of listeriosis have emerged as an important foodborne illness of global concern [1, 2]. It is a major public health problem for at-risk groups, causing high morbidity and mortality. Pregnant women had a 20-times more risk of developing listeriosis compared to the general population [3]. Studies indicated that LM is responsible for spontaneous abortion, stillbirth, and preterm delivery in pregnant women [3, 4]. The infection can be transmitted from mother to growing fetus through placenta following maternal septicemia, or by the ingestion of amniotic fluid and passage through infected birth canal [5]. Invasive listeriosis is usually associated with pregnant women, infants, neonates, organ transplant recipients, HIV, and cancer patients, and the clinical presentations of invasive listeriosis include septicemia, meningitis, meningoencephalitis, and gastroenteritis [6, 7].

The predilection of LM in the feto-maternal unit contributes to fatal outcomes in the fetus and/or mother. However, the exact mechanism often remains unexplained [8]. Listeriosis is usually treated by a combination of ampicillin and aminoglycoside [9, 10]. However, LM isolates had shown variable responses to commonly prescribed antibiotics [11]. Studies on the magnitude and antimicrobial susceptibility pattern of human listeriosis are scarce in most African countries including Ethiopia. Thus, we aimed to isolate and characterize LM in terms of antibiogram and biofilm formation.

## Methods

### Study setting

A prospective cross-sectional study was conducted at JUMC from June 1, 2019, to August 30, 2019. JUMC is one of the teaching and referral hospitals in the South Western part of Ethiopia, providing different services for a catchment population of about 15 million people. JUMC has 659 beds out of which 52 beds in maternity and 60 beds in the gynecology and obstetrics ward. The medical center admits more than 20,000 patients and its yearly outpatient visits is more than 170,000 patient [12].

### Eligibility criteria

Pregnant women with fever, women having spontaneous abortion, and women who had preterm delivery were included in the study. While pregnant women who were on antibiotics in the past 2 weeks before the study and women having safe abortion care were excluded from the study.

### Data collection and culture

A total of 144 women who fulfill the inclusion criteria, and willing to participate in the study were recruited. Trained midwives conducted a face-to-face interview using the structured questionnaire, and collected maternal blood and cord blood specimens. There were 138 maternal blood and 6 cord blood samples collected from 144 women. 5 to 10 ml of venous or cord blood was directly inoculated into 50 ml of tryptic soy broth (difco, USA) with 0.6% yeast extract (TSBYE) (Oxoid, England) and incubated at 35 °C for 24 h. Subculture was made immediately after the first sight of growth noticed onto Listeria selective agar (LSA) medium (Himedia, India). The results were reported as negative if samples showed no evidence of growth on TSBYE for 7 days [13].

### Isolation of *L. monocytogenes*

*L. monocytogenes* typically grows as a small yellow colony on LSA and grey to white beta-hemolytic colonies on 5% sheep blood agar (Himedia, India). Preliminary identification of LM was done by Gram staining, hemolysis pattern on 5% sheep blood agar, and observation of its tumbling motility in a wet mount light microscopy after overnight incubation of typical colonies inoculated in TSBYE at 25 °C and 37 °C. Furthermore, Catalase, Oxidase, CAMP factor test, and sugars fermentation (mannitol, maltose, dextrose, sucrose, lactose, and rhamnose) was performed for species identification and confirmation of LM [14–16].

### Antimicrobial susceptibility testing

Three to five pure colonies of LM from overnight grown culture were suspended in sterile normal saline. The turbidity of suspension was checked against the 0.5 McFarland standard. Antimicrobial susceptibility testing (AST) was performed by Kirby Bauer disk diffusion technique on Muller Hinton Agar (MHA) supplemented with fresh 5% sheep blood (Himedia, India). Antibiotics tested include: Doxycycline (30 μg), Penicillin G (10 μg), Cotrimoxazole (25 μg), Ampicillin (10 μg), Erythromycin (15 μg), Clindamycin (2 μg), Ciprofloxacin (5 μg), Gentamicin (10 μg), Rifampicin (5 μg) and Meropenem (10 μg)). Suspended isolates were inoculated on MHA supplemented with 5% sheep blood, antibiotic discs placed firmly and incubated at 37^0^ C for 24 h. Zone of inhibition was measured and interpreted according to CLSI 2016 [17] and EUCAST guideline for LM [18].

### Detection of biofilm production

Microtiter plate assay is the most frequently used method to determine biofilm production. The protocol used was adapted from Djordjevic et.al [19] and the result was interpreted as per [20].

### Statistical analysis

Data were checked for completeness, coded, and entered into Epi-data version 3.1, and exported to SPSS v. 25.0 for analysis. Binary logistic regression was conducted for socio-demographic and clinical variables against LM positivity. Nonparametric correlation analysis was done for isolates against the number of antibiotics resistant and susceptible. *P*-value < 0.05 was considered statistically significant.

### Quality assurance

The prepared questionnaire was evaluated by obstetricians. 5% of the questionnaire was pretested at Shenan Gibe hospital, the nearby hospital in Jimma town. The training was provided for data collectors, and the collected data were checked for completeness in the field. LM (ATCC 19115) was used as the reference strain for *Listeria monocytogenes*.

## Results

### Socio-demographic characteristics

A total of 144 women participated in this study. The mean age of women was 26.48 ± 5.03 years. The majority (59% (85/144)) of women were aged between 25 and 34 years old. More than 2/3rd of study participants had formal education, and 73.6% (*n* = 106/144) of women were housewives (Table 1.). LM was isolated in 5.56% (8/144) of all study participants. Seven of the isolates were recovered from 138 maternal blood (5.07%). One of the six cord blood (16.67%) was positive, indicating the rate of transplacental transmission at JUMC.

**Table 1:** Bivariate analysis of socio-demographic characteristics among study participants at JUMC, June to August 30, 2019, southwest Ethiopia

Socio-demographic characteristics (*n* = 144)		Frequency (%)	*L. monocytogenes*		*P* value	95% C.I^a^
			Yes (%)	No (%)		
Age of women	18–24	45 (31.3)	1 (2.2)	44 (97.8)	1	
	25–34	85 (59.0)	6 (7.1)	79 (92.9)	0.4	3.385 (0.198, 57.931)
	35–45	14 (9.7)	1 (7.1)	13 (92.9)	0.991	1.013 (0.113, 9.111)
Residence	Urban	93 (64.6)	5 (5.4)	88 (94.6)	0.899	1.1 (0.252, 4.803)
	Rural	51 (35.4)	3 (5.9)	48 (94.1)		
Formal education	No	48 (33.3)	2 (4.2)	46 (95.8)	0.609	1.533 (0.298, 7.899)
	Yes	96 (66.7)	6 (6.2)	90 (93.8)	1	
Occupation (Housewife)	Yes	106 (73.6)	7 (6.6)	99 (93.4)	1	
	No	38 (26.4)	1 (2.6)	37 (97.4)	0.376	2.616 (0.311, 21.994)

^a^C.I. stands for the confidence interval

### Clinical sign and symptoms

Among the study participants, 44.4% (*n* = 64)) had preterm delivery, 34% (*n* = 49) had ongoing pregnancy, and 21.6% (*n* = 31)) had a fetal loss. Fever (*n* = 100) and headache (*n* = 62) the most frequent symptoms noted. Of the 8 isolates, the majority were recovered from women with fever (*n* = 7) and headache (*n* = 5). More than 32% (*n* = 47) of women experienced bad obstetrics history (preterm delivery, stillbirth, and spontaneous abortion) in the past, at least once in their lifetime. Underlying medical conditions were also noted in pregnant women; HIV/AIDS (*n* = 5), chronic hepatitis (*n* = 4), hypertension (*n* = 3), and heart disease (*n* = 2). However, LM was not recovered in any of the women with underlying diseases. LM isolation rate was relatively higher (9.7%) among women with fetal loss, and women with preterm delivery (6.25%). None of the clinical characteristics were found correlated with LM recovery (Table 2.).

**Table 2:** Bivariate analysis of clinical characteristics and LM positivity from pregnant women at JUMC, June to August 30, 2019, southwest Ethiopia

Clinical variables (*n* = 144)	No (%)	*L. monocytogenes*		*P*-value	95% C.I.^a^
		Yes (%)	No (%)		
Clinical sign and symptoms					
Fever	100 (69.4)	7 (7.0)	93 (93.0)	0.279	0.309 (0.037, 2.590)
Headache	62 (43.1)	5 (8.1)	57 (91.9)	0.265	0.433 (0.099, 1.885)
Gastroenteritis	12 (8.3)	0 (0)	12 (100)	0.999	0.000
Vomiting	15 (10.4)	0 (0)	15 (100)	0.999	0.000
Gestational age					
< 28 weeks	28 (19.4)	1 (3.6)	27 (96.4)		1
≥ 28 weeks	116 (80.6)	7 (6.0)	109 (94.0)	0.614	0.577 (0.068, 4.888)
had past “bad obstetrics history”	47 (32.6)	4 (8.5)	93 (91.5)	0.291	0.462 (0.110, 1.936)
No past “bad obstetrics history”	97 (67.4)	4 (4.1)	43 (95.9)		1

^a^C.I. stands for the confidence interval

“ ” women with recurrent spontaneous abortions, history of fetal loss or history of preterm delivery

### Antimicrobial susceptibility of *L. monocytogenes*

**Fig. 1:**
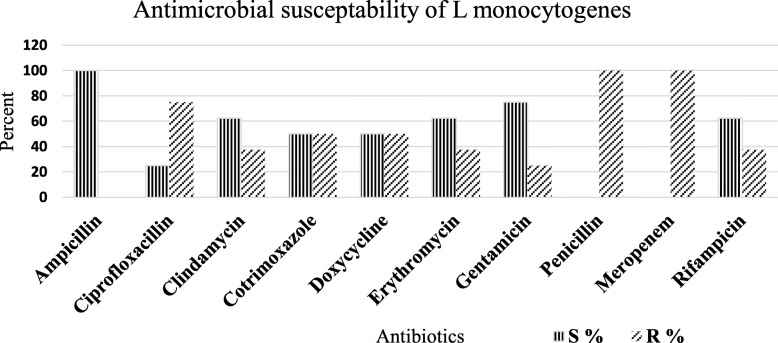
Antibiogram of LM among women with fever, women with preterm delivery, and women with a history of fetal loss at JUMC, June to August 30, 2019, Southwest Ethiopia

All of the isolates were shown resistant (100%) for meropenem and penicillin. LM isolates also showed a relatively decreasing level of resistance to ciprofloxacin (*n* = 6, 75%), cotrimoxazole or doxycycline (*n* = 4, 50%), erythromycin or clindamycin (*n* = 3, 37.5%), and gentamicin (*n* = 2, 25%). Ampicillin was found the choice of antibiotics, susceptible to all isolates followed by gentamicin (75%), rifampicin (62.5%), erythromycin (62.5%), and clindamycin (62.5%) (Fig. 1).

### Biofilm production

The microtiter plate assay showed that all the isolates were biofilm former. Isolates OD value was cross-tabulated against the number of antibiotics (Table 3).

**Table 3:** OD value of isolates with the number of antibiotics susceptible and resistant

Isolate	OD of Isolates	No of antibiotics	
		Susceptible	Resistant
Isolate 1	0.0926	7	3
Isolate 2	0.1270	5	5
Isolate 3	0.0705	3	7
Isolate 4	0.0895	5	5
Isolate 5	0.0646	5	5
Isolate 6	0.1702	4	6
Isolate 7	0.2081	3	7
Isolate 8	0.3857	2	8

The Spearman correlation analysis of OD value of biofilm assay and the number of antibiotic-resistant for LM showed an insignificant association (*p* > 0.05).

## Discussion

Pregnancy-related listeriosis is an important public health concern of feto-maternal units due to the high morbidity and mortality to the fetus and or mother [8, 21]. In the current study, LM was isolated in 5.56% (8/144) of the study participants; which was in agreement with findings in Germany (3.3%) [22], Colorado, USA (2.5%) [23], and Indonesia (9%) [13]. Similarly, our finding was in line with African studies; west Africa (4.6%) [24], Nigeria (8.04%) [25], and the previous study in Tigray, Ethiopia (8.5%) [26]. These consistent findings could suggest the global importance of pregnancy-associated listeriosis irrespective nation’s developmental status. On other hand, our finding was higher as compared to India (0.32%) [27] and India (0.81%) [28], and Tanzania (0.68%) [29]. The discrepancy could be explained by the variation in the laboratory method used, population difference, and exposure variability.

Our isolates were completely resistant to meropenem and penicillin, followed by ciprofloxacin resistance (75%) (Fig. 1.). The increased resistance to penicillin in this study was similar to the previous study in Tigray, Ethiopia [26]. This directs the essence of an antibiogram for on-time and accurate treatment of pregnant women and their fetuses, as penicillin is the drug of choice. Ampicillin was found the choice of antibiotics in our study, as do findings in India [28], Poland [30] Iran [31], and Brazil [9]. The resistance to penicillin (100%), cotrimoxazole (50%), and gentamicin (25%) in the current study (Fig. 1.) were higher compared to 28.6% resistance to penicillin and no resistance to cotrimoxazole and gentamicin reported in India [32]. The above findings presented the emergence of antibiotic-resistant strains with a varying degree of resistance [11, 26, 31, 32]. These could show the potential threat to public health by antibiotic resistance LM strains and implicates the need for AST before any antibiotic administration to pregnancy-associated listeriosis.

This study noted that all of the isolates were biofilm producers, which was in agreement with the study in Poland [30]. The biofilm production might offer an opportunity to persist on food processing devices and medical devices like catheters and devices related to delivery [30] increase the risk of getting infected [33]. Our study also investigated that the biofilm production capacity of LM was found not associated with drug resistance. However, the higher resistance to penicillin, meropenem, and ciprofloxacin might be connected with biofilm formation [34].

We isolated and characterized LM in terms of antibiogram and biofilm formability among pregnant women with fever, women with preterm delivery, and women with a history of fetal loss at JUMC, Ethiopia for the first time. Due to the nature of the study, we were limited to include asymptomatic pregnant women and other at-high risk groups. We were also unable to assess molecular grounds why isolates were ampicillin susceptible but resistant to penicillin and carbapenem due to limited resources. The high prevalence of pregnancy-related listeriosis in the current study setting appears that implementation of educational programs targeting risk reduction and more studies to identify sources of LM are warranted. Increased resistance of LM to penicillin, meropenem, ciprofloxacin, cotrimoxazole, and doxycycline; indicated that the choice of antibiotics should be after antimicrobial susceptibility testing.

## Data Availability

The datasets used or analyzed during the current study are available from the corresponding author on reasonable request.
